# Kynurenic Acid and Its Analogs Are Beneficial Physiologic Attenuators in Bdelloid Rotifers

**DOI:** 10.3390/molecules24112171

**Published:** 2019-06-10

**Authors:** Zsolt Datki, Zita Galik-Olah, Zsuzsanna Bohar, Denes Zadori, Ferenc Fulop, Istvan Szatmari, Bence Galik, Janos Kalman, Laszlo Vecsei

**Affiliations:** 1 Department of Psychiatry, Faculty of Medicine, University of Szeged, Kalvaria sgt. 57, H-6725 Szeged, Hungary; olah.zita.87@gmail.com (Z.G.-O.), kalman.janos@med.u-szeged.hu (J.K.); 2 MTA-SZTE Neuroscience Research Group, Semmelweis u. 6, H-6725 Szeged, Hungary; zsuzsanna.bohar@gmail.com (Z.B.); vecsei.laszlo@med.u-szeged.hu (L.V.); 3 Department of Neurology, Interdisciplinary Excellence Centre, Faculty of Medicine, University of Szeged, Semmelweis u. 6, H-6725 Szeged, Hungary; zadori.denes@med.u-szeged.hu; 4 Institute of Pharmaceutical Chemistry, Interdisciplinary Excellence Centre, Faculty of Pharmacy, University of Szeged, Eötvös u. 6, H-6720 Szeged, Hungary; fulop@pharm.u-szeged.hu (F.F.), Szatmari.Istvan@pharm.u-szeged.hu (I.S.); 5 MTA-SZTE Stereochemistry Research Group Eötvös u. 6, H-6720 Szeged, Hungary; 6 Department of Clinical Molecular Biology, Medical University of Bialystok, ul.Jana Kilinskiego 1, 15-089 Bialystok, Poland; bence.galik@umb.edu.pl

**Keywords:** kynurenic acid, metabolism, physiology, bdelloid rotifer, survival, mastax

## Abstract

The in vivo investigation of kynurenic acid (KYNA) and its analogs is one of the recent exciting topics in pharmacology. In the current study we assessed the biological effects of these molecules on bdelloid rotifers (*Philodina acuticornis* and *Adineta vaga*) by monitoring changes in their survival and phenotypical characteristics. In addition to longitudinal (slowly changing) markers (survival, number of rotifers alive and body size index), some dynamic (quickly responding) ones (cellular reduction capacity and mastax contraction frequency) were measured as well. KYNA and its analogs increased longevity, reproduction and growth, whereas reduction capacity and energy-dependent muscular activity decreased conversely. We found that spermidine, a calorie restriction mimetic, exerted similar changes in the applied micro-invertebrates. This characterized systemic profile evoked by the above-mentioned compounds was named beneficial physiologic attenuation. In reference experiments, using a stimulator (cyclic adenosine monophosphate) and a toxin (sodium azide), all parameters changed in the same direction (positively or negatively, respectively), as expected. The currently described adaptive phenomenon in bdelloid rotifers may provide holistic perspectives in translational research.

## 1. Introduction

The kynurenine (KYN) pathway plays an important role in several biological systems affected by aging, via modulating the cellular changes related to oxidative stress, mitochondrial dysfunction, inflammation, cognitive and immune responses [1]. KYNs are endogenous intermediates of L-tryptophan degradation, a pathway in which serotonin, nicotinic acid and its derivatives, e.g., nicotinamide adenine dinucleotide (NAD^+^) is produced [2]. KYNs became the focus of scientific attention in the 1980s with the discovery of some intermediates having primary neuroactive and protective properties [3] as broad-spectrum ionotropic glutamate receptor antagonists [4,5]. Elevated concentrations of kynurenic acid (KYNA) in cerebrospinal fluid were observed in aged populations [6], while in the invertebrate *Caenorhabditis elegans* the depletion of KYNA improved learning and memory [7]. This molecule inhibits the overexcitation of glutamatergic transmission; furthermore, it may influence nutrition and metabolism, via the regulation of food-dependent behavioral plasticity [8,9]. In *C. elegans* the level of serotonin increases during feeding behavior, even in the absence of food. The tryptophan hydroxylase-mutant animals, which thus lack serotonin, showed reduced feeding rates even in the presence of food [10], proving a key role of the serotonergic system in the regulation of metabolism.

The well-known rotifer-aging model [11], also used in our laboratory, applies to bdelloid species, which have a complex nervous system mainly based on serotonergic regulation [12,13]. Thus, they seem to be adequate for investigations of the relationship between KYNA, its analogs and physiology. Bdelloid rotifers are widely used microinvertebrate models in the research fields of ecotoxicology [14] and aging [15]. They are eutelic (grown by hypertrophy) animals with well-defined viability markers and anatomical parameters. In our previous work [16], we developed a complex in vivo experimental viability assay for high-throughput screening, by measuring the survival, number of rotifers alive (NRA), body size index (BSI), cellular reduction capacity (CRC) and mastax contraction frequency (MCF). All these characteristics were applied in the current experiments as well.

**Figure 1:**
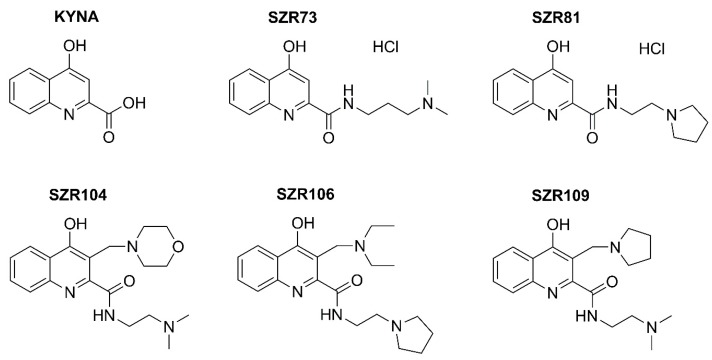
Formulas of kynurenic acid (KYNA) and its SZR analogs.

As KYNA does not penetrate the blood-brain-barrier, several analogs have been synthesized to improve the efficacy in preclinical studies [17,18]. Our aims were to test KNYA and its five analogs (Figure 1) for the first time in rotifers and to merge the previous separate assays into a comprehensive physiological model for further complex screening of bioactive and pharmaceutical molecules. The elevated survival of individuals in the population and the reverse change of the NRA-BSI pair compared to the CRC-MCF pair showed that KYNA and some analogs had special, beneficial physiologic attenuation effects on our microinvertebrates. Survival, NRA and BSI are longitudinal responding markers of the status of individual physiology in contrast to CRC and MCF, which are quickly changing ones. Our work presents a new systemic property, a holistic influence of KYNA and its SZR73 and SZR81 derivatives, in increasing survival, reproduction and growth in contrast to cellular reduction and mastax activity in bdelloids.

## 2. Results and Discussion

KYNA has a key role in numerous biological species and their environment. The natural abundance of this biomolecule is essential in plants [19], in animal feed [20], in amphibians as pheromones [21] and moreover in fish as lymphocyte-activators [22] with potential aggravating properties [23].

**Figure 2:**
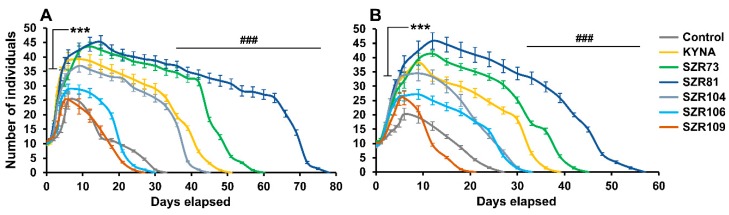
Survival kinetics of rotifer populations treated (100 µM) with KYNA or its analogs. The number and survival of individuals significantly increased in the three treated groups compared to untreated controls. KYNA and its SZR73 and SZR81 analogs had the most beneficial effect on both *Philodina acuticornis* (**A**) and *Adineta vaga* (**B**) species. The two different bdelloid rotifer genera show similar kinetics under the same treatments. The error bars represent SEM. One-way ANOVA with Bonferroni *post hoc* test was used for statistical analysis, the level of significance is ***; ### *p* < 0.001 (*, significant difference in number of rotifers from untreated controls on day nine; # significant difference from untreated control in survival of animals; *n* = 12 wells).

Rotifers are recent models that have gained scientific acceptance in pharmaceutical screening [24,25]. Our aim was to study the physiological impacts of KYNA and its analogs (SZRs) on *Philodina acuticornis* and *Adineta vaga* in a holistic approach. First, we investigated the effects of these molecules on the survival of bdelloids. Population kinetics, influenced by the analogs, were measured after one-time feeding in a self-limiting system. We found that the survival of *P. acuticornis* significantly increased in the presence (100 µM; Figure 2A) of KYNA (155%), SZR73 (182%), SZR81 (236%) or SZR104 (136%) compared to the untreated control (100%). These molecules significantly elevated the number of individuals, where the level of significance was determined based on the effect of sodium-azide (NaN_3_; 100 µM) toxin, which induced the shortest survival (33%; nine days) in our experiments. The most effective agent was SZR81. In *A. vaga*, the efficacy of the drugs was measured under the same experimental conditions. The *A. vaga* populations exhibited significant survival in the presence (100 µM; Figure 2B) of KYNA (144%), SZR73 (167%), SZR81 (211%) or SZR104 (122%), after one-time feeding. On day nine, the number of rotifers was significantly higher in KYNA, SZR73, SZR81 or SZR104 treated groups than in the untreated control. SZR81 also showed the most powerful impact in these species. This suggested that KYNA and its analogs had positive effects on survival (Figure 2), except for SZR106 (*P. acuticornis*: 91%) and SZR109 (*P. acuticornis*: 82%; *A. vaga*: 78%). Although their efficacy was different, the tendencies did not show any species specificity. 

**Figure 3:**
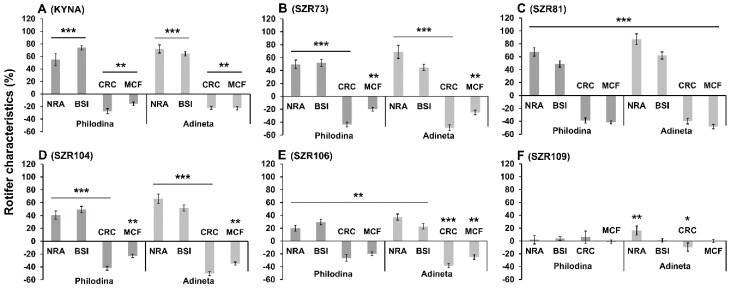
Biological characteristics of rotifers under treatment (100 µM) with KYNA or its analogs showing contradictory profiles. On day six, three experimental- (number of rotifers alive (NRA), body size index (BSI), mastax contraction frequency (MCF)) and one chemical cellular reduction capacity (CRC) parameter of individual rotifers and the populations were monitored. KYNA (**A**) and its SZR analogs (**B**–**E**) had antagonistic profiles for the following characteristics: NRA-BSI compared to CRC-MCF pairs, with the exception of SZR109 (**F**). Both *Philodina* and *Adineta* species show significant differences from their untreated controls (showing as 0 value on the *x* axis in graphs). The error bars represent SEM. One-way ANOVA with Bonferroni *post hoc* test was used for statistical analysis, the levels of significance are * *p* ≤ 0.05, ** *p* ≤ 0.01 and *** *p* ≤ 0.001. (*, significant difference from untreated controls, where the corresponding data of 100% is the 0 value on the *x* axis); NRA, *n* = 24 wells; BSI, *n* = 50 randomly selected individuals from 24 wells; CRC, *n* = 24 wells; MCF, *n* = 50 randomly selected individuals from 24 wells).

Thereafter, we studied how these advantageous molecules influenced the physiological and viability characteristics of microinvertebrates. In our experiments, four previously described parameters (NRA, BSI, CRC and MCF) [16,26] were measured on day six, since the survival curves of the untreated control population (Figure 3) reached their maximum number of rotifers then. The above-mentioned values, related to physiologically acute reactions, were presented relative to the untreated control values (100%), which were converted to the zero point on the “x” axis of the graphs. In the presence of the basic molecule, KYNA (Figure 3A), the same pronounced characteristic profile was observed in both animal species. We explored how the analogs influenced the rotifers in comparison to the untreated controls. The significant reverse change of the NRA-BSI pair compared to the CRC-MCF pair showed that KYNA and some analogs have an interesting contradictory effect. SZR73 (Figure 3B) and SZR81 (Figure 3C) significantly enhanced these opposing effects. The degree of change induced by SZR104 (Figure 3D) was approximately the same as that of KYNA treatment. The SZR106 (Figure 3E) effect showed a similar profile to the base molecule where all the measured parameters significantly changed. We found that SZR109 (Figure 3F) was the least effective; only the NRA and CRC of *A. vaga* populations exhibited significant alterations compared to the controls, but these changes were not so pronounced.

The most common property of the above-described in vivo physiological parameters was the alteration in metabolism [15,27], since all these processes had relatively high-energy demands [28,29]. The NRA was influenced by the fecundity of the individuals in the population, while the BSI was the result of cellular growth in the euthelic rotifers [30]. Both characteristics are associated with active protein synthesis, which is the basis of anabolic processes [31]. The survival of individuals in the population, together with the elevation of NRA and BSI, adequately imply the great viability of the species, which was positively enhanced by KYNA and some of its analogs. CRC referred to the cellular reduction capacity in the population, which is a NADH dependent biochemical reaction [32]. According to the academic literature, a certain degree of attenuation in these processes may have beneficial effects on viability [33,34]. Decreasing cellular NADH levels is a protective approach in biological systems [35,36]. As another rotifer-related indicator, active muscle function was necessary for the maintenance of mastax activity [16], which was one of the most energy-consuming operations in these model animals [31]. In our experiments, the CRC and MCF were reduced in individuals where the NRA and BSI were elevated in the presence of KYNA and related molecules. The reduced parameters may indicate a slowed metabolism, being the molecular basis of the calorie restriction-induced positive impacts [37,38]. In rotifers, the calorie restriction and chemical redox modulation were able to synergistically enhance the physiological parameters and longevity [15]. Our experimental (survival, NRA, BSI and MCF) and redox (CRC) parameters were appropriate to assess the in vivo changes in metabolism.

**Figure 4:**
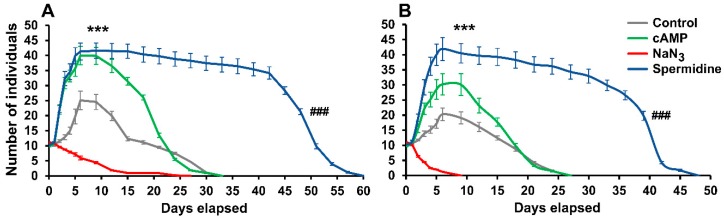
Survival kinetics of rotifer populations treated (100 µM) with stimulator, toxin or calorie restriction mimetic. The number and survival of individuals significantly increased under spermidine treatment, compared to untreated controls. The cyclic adenosine monophosphate (cAMP) had a positive effect on the reproduction of individuals; however, it had no pronounced influence on the survival of animals. NaN_3_ was toxic to both *Philodina acuticornis* (**A**) and *Adineta vaga* (**B**) species. The two different rotifer genera show similar kinetics under the same treatments. The error bars represent SEM. One-way ANOVA with Bonferroni *post hoc* test was used for statistical analysis, the level of significance is ***; ### *p* < 0.001 (*, significant difference in number of rotifers from untreated controls on day nine; # significant difference from untreated controls in survival of animals; *n* = 12 wells).

**Figure 5:**
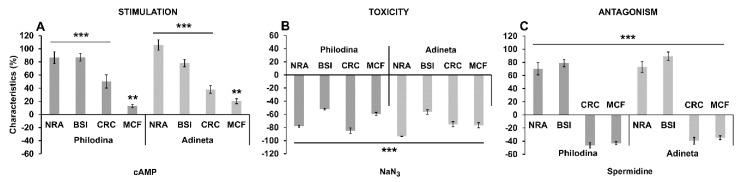
Biological characteristics of rotifers treated (100 µM) with stimulator, toxin or calorie restriction mimetic having contradictory profile. On day six, three experimental- (NRA, BSI, MCF) and one chemical (CRC) parameter of individual rotifers and populations were monitored. All parameters show significant differences from their untreated controls (shown as zero value on the *x* axis in graphs) in a positive direction evoked by cAMP (**A**) in contrast to NaN_3_ (**B**). Spermidine (**C**) has an antagonistic profile related to the NRA-BSI compared to CRC-MCF pairs. These three types of treatments show different biological profiles (stimulation, toxicity and antagonism) in our rotifer models. The error bars represent SEM. One-way ANOVA with Bonferroni *post hoc* test was used for statistical analysis, the levels of significance are * *p* ≤ 0.05, ** *p* ≤ 0.01 and *** *p* ≤ 0.001. (*, significant difference from untreated controls, where the corresponding data of 100% is the zero value on the *x* axis); NRA, *n* = 24 wells; BSI, *n* = 50 randomly selected individuals from 24 wells; CRC, *n* = 24 wells; MCF, *n* = 50 randomly selected individuals from 24 wells).

In order to gain a deeper understanding of the inverse alteration profile of the investigated KYNA-molecules, we examined which reference treatments showed the same pattern regarding rotifer physiology (Figure 4 and Figure 5). A stimulator, a toxin and a calorie restriction mimetic were applied in our experiments. Cyclic adenosine monophosphate (cAMP; 100 µM), as a potential stimulator [39], had no effect on survival (*P. acuticornis*: 99%; *A. vaga*: 98%), while at the same time the number of rotifers significantly increased (Figure 4). However, the NaN_3_ toxin [40] decreased survival (*P. acuticornis*: 82%; *A. vaga*: 33%) and the total number of individuals. The reference point (Figure 2 and Figure 4), day nine, was the lowest survival measured in the *A. vaga* population (Figure 4B). Spermidine (100 µM), a well-known calorie restriction mimetic [41], significantly increased survival (*P. acuticornis*: 181%; *A. vaga*: 179%) and other parameters, similarly to KYNA and some of its derivatives. Henceforth, we tested how these profile-reference molecules affected the physiological characteristics of rotifers. All four parameters were positively elevated by cAMP induced stimulation (Figure 5A) in both species. In NaN_3_ toxicity (Figure 5B) measurements, the parameters of interest showed uniform decrease. Antagonistic profiles were observed in spermidine-treated animals (Figure 5C), similarly to groups treated with KYNA or its analogs. The elevated NRA-BSI and the decreased CRC-MCF pairs appeared simultaneously with prolonged survival. The significant increase of slow-changing, time-consuming physiological markers (survival, NRA, BSI), together with the alteration of dynamic changing indicators (CRC and MCF) formed a specific physiological profile that we named beneficial physiologic attenuation (BPA). ‘Beneficial’ refers to the positive changes in survival, NRA and BSI, while ‘attenuation’ means the non-toxic type of decrease as in the case of CRC and MCF. The measured characteristics describe the energy-dependent life quality, covering both the chronic and acute alterations. Since, the exact mechanism of action of KYNA and its analogs is as yet unclear in rotifers, our objective in this present work was to describe the BPA phenomenon. We assumed that its molecular background might be related to serotonergic regulation [11] and redox modulation [15,38] in these particular bdelloids.

**Figure 6:**
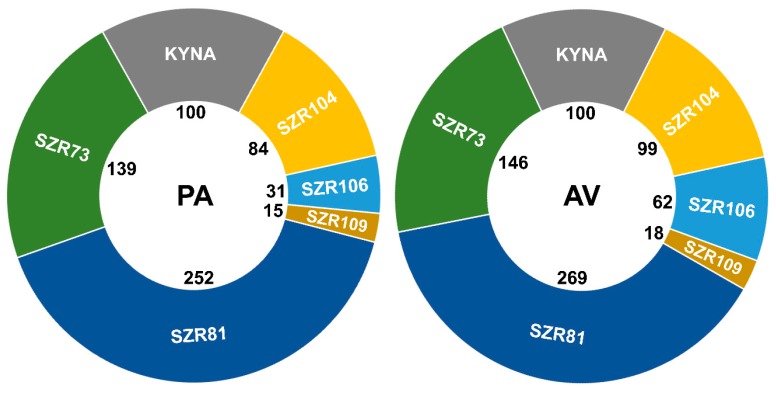
Beneficial physiologic attenuation index (BPA_i_) of KYNA or its analogs (100 µM). KYNA is the reference molecule (100% BPA) for SZR analogs. SZR73 and SZR81 were the best compounds based on their physiological influences on *Philodina acuticornis* (PA) and *Adineta vaga* (AV) species. All monitored rotifer-specific parameters were integrated into a comprehensive mathematical formula, calculating percentile beneficial physiologic attenuation (BPA) data.

The BPA model includes different changes, hence, we developed a summary mathematical formula, called the BPA index (BPAi). The parameters described above were integrated into BPAi, thus, it was possible to compare the molecules which potentially modulate physiological processes. BPAi is a relative number, presented in percentage value (Figure 6). The experimental reference molecule for the BPA model was spermidine. BPAi values of this polyamine compound relative to KYNA (100%) were 208% in *P. acuticornis* and 206% in *A. vaga*. These species showed 99% similarity regarding spermidine efficacy compared to KYNA. The analogs were compared to KYNA (Figure 6).

Our present work described a novel and complex application of rotifers using them as in vivo models to investigate the indicators of metabolic modulation. We demonstrated the impacts of KYNA and its SZR-analogs in a system applying bdelloid rotifers, which showed the experimental efficacy of these agents on microscopic living creatures.

## 3. Materials and Methods 

### 3.1. Materials

The analogs (SZR73, SZR81, SZR104, SZR106, SZR109) of KYNA (cat# K3375, Sigma Aldrich, St. Louis, MO, USA) were prepared in the Institute of Pharmaceutical Chemistry, Interdisciplinary Excellence Centre, University of Szeged, Szeged, Hungary. In our system we separately applied the following reference molecules: cAMP (cat# A9501, Sigma Aldrich) as a stimulant, NaN_3_ (cat# 822335, Merck Kenilworth, NJ, USA) as a toxin and spermidine (cat# S2626, Sigma Aldrich) as a well-known calorie restriction mimetic.

#### 3.1.1. The Invertebrate Models

The experiments were performed on invertebrate bdelloid rotifers *P. acuticornis* and *A. vaga*; therefore, according to the current international regulations, no specific ethical permission was needed. They were obtained from a Hungarian aquavaristique with origins from an agricultural farm in Szarvas, Hungary. The species have been maintained in a standard laboratory environment for six years. Our measurements were carried out in accordance with globally accepted norms: Animals (Scientific Procedures) Act, 1986, associated guidelines, EU Directive 2010/63/EU for animal experiments, and the National Institutes of Health guide for the care and use of Laboratory animals (NIH Publications No. 8023, revised 1978). Animal studies comply with the ARRIVE guidelines.

The rotifers were cultured following the methods in our previous publication [16] with the following modification: The fibrillar alga-based environmental matrix was changed to ethanol- and distilled water-washed organic hemp yarn (Emil Lux GMBH & Co. KG, Wermelskirchen, Germany).

#### 3.1.2. Treatment and Monitoring

Populations were harvested based on our previous study [16] in half-area 96 well-plates (cat# 3695, Costar, Corning Inc., New York, NY, USA) for the experiments. The flasks were placed at –75 °C for 3 min for rapid cooling and release of attached live rotifers from the hemp-matrix. The cooled medium (~ 4 °C) was poured off (together with numbed rotifers) and the flasks were washed again with cooled (4 °C) standard medium (mg/L): Ca^2+^ 31.05; Mg^2+^ 17.6; Na^+^ 0.9; K^+^ 0.25; Fe^2+^ 0.001; HCO_3_^−^ 153.097; SO_4_^−^ 3; Cl^−^ 0.8; F^−^ 0.02; H_2_SiO_3_ 3.3 (pH = 7.5). The healthy animals were allowed to attach to the well bottom. The starting number of rotifers (mixed ages/sizes) per well (100 µL) was 10 ± 2. Each treatment agent was dissolved (stock solution; 10 mM) in standard medium [16]. At the beginning of the measurements, we applied the feeding solution (600 µg/mL homogenate), which was heat-inactivated and filtered (Whatman filter with 10 um pore, cat no: 6728-5100) baker’s yeast (EU-standard granulated instant form, cat# 2-01-420674/001-Z12180/HU). In all the treatments (final drug concentration: 100 µM/compound), the one-time treatment and feeding were administered simultaneously. To characterize and to compare the effects of the compounds on rotifer populations and individuals, five assays were used: Survival of individuals in population (*n* = 12 wells)*,* NRA (*n* = 24 wells), BSI (*n* = 50 randomly selected individuals from 24 wells), CRC (*n* = 24 wells) and MCF (*n* = 50 randomly selected individuals from 24 wells). The comparison reference point in experiments on survival kinetics was day nine, which was the shortest survival time measured in the *A. vaga* population (Figure 4B). This relative time point was applied uniformly during all survival experiments. These recordings are longitudinal in contrast to the other characteristics, which are the end point (day six) experiments. Usually, day six is the maximum point of the kinetic curve of controls followed by the decrease of NRA in this self-limiting closed system. During survival and NRA experiments we monitored the animals daily using an inverted light microscope (Leitz Labovert FS, Germany). Active motion and general movement within the body defined the living individuals. The rotifers were considered dead after loss of the telescopic reflex, abnormal morphology of the body and the appearance of fragmentation or amorphous granules in the soma. The BSI was monitored and calculated daily by the following formula: BSI (%) = maximal ’length × width’ of body (µm). Animals were photographed (digital camera) under the microscope by taking serial images per entity. The CRC was detected by the EZ4U Cell Proliferation Assay (cat# BI-5000, Biomedica, Vienna). To avoid toxicity, 20× diluted XTT solution was used. The plates were incubated for 24 h without direct light at room temperature. The sum reduction capacity of rotifers was measured. The absorbance was measured by a microplate-reader set at 492 nm with 630 nm as a reference. The readings were normalized to the number of animals/well. To evaluate the energy dependent muscular activity of the individuals we recorded the MCF (contraction per sec). The mastax is part of the digestive system and its function is to shred the food by periodically opening and closing.

#### 3.1.3. Statistics

To calculate the percentile value of the BPA index (BPA_i_%), the following formula was used: [(T_max_*√∑R)]*[NRA%(>100) + BSI%(>100)]D6/[CRC%(<100) + MCF%(<100)]D_6_. T_max_ is the maximal time of survival of individuals in the population and the ‘R’ is the number of all rotifers until T_max_. Statistical analysis was performed with SPSS 23.0 (SPSS Inc, Chicago, IL, USA) using one-way ANOVA with Bonferroni *post hoc* test. The error bars represent the standard error of the mean (SEM). The different levels of significance are indicated as follows: * *p* ≤ 0.05, ** *p* ≤ 0.01 and *** *p* ≤ 0.001.
